# Our Contemporaries

**Published:** 1917-05

**Authors:** 


### OUR CONTEMPORARIES

The Old Dominion Journal of Medicine and Surgery, of Richmond, has been discontinued. It was a good journal which we shall miss.

The Pacific Medical Journal of April devotes many pages to newspaper and true accounts of malpractice suits. Those interested in medico-legal matters, especially professional self-protection, should secure this issue.

The Critic & Guide of April devotes much space to the discussion of war, including contributions by several pacifists. While there are some general principles with which we agree, when it comes to the relative merits of peace and war, we seldom encounter matter with which we disagree so heartily and in detail. We say this in a spirit of friendship.

The National Assn. for the Study and Prevention of Tuberculosis will begin the publication of a monthly journal devoted to tuberculosis. Dr. Edward R. Baldwin of Saranac Lake will be editor-in-chief.

The Annals of Medical History will appear as a quarterly, of large size, at $6 per year, published by Paul B. Hoeber of N. Y., edited by Dr. Francis R. Packard of Philadelphia, beginning this spring.

